# Short-term survival rates of 1397 horses referred for colic from 2010 to 2018

**DOI:** 10.1186/s13028-022-00631-4

**Published:** 2022-05-07

**Authors:** Emma Dybkjær, Kirstine Fleng Steffensen, Marie Louise Honoré, Mathias Ankjær Dinesen, Mogens Teken Christophersen, Tina Holberg Pihl

**Affiliations:** Department of Veterinary Clinical Sciences, Faculty of Health and Medical Sciences, University of Copenhagen, Agrovej 8, 2630 Taastrup, Denmark

**Keywords:** Colic, Diagnosis, Equine, Gastro-intestinal, Medical, Outcome, Surgery

## Abstract

**Background:**

Up-to-date and hospital-specific knowledge of prognoses for horses with various forms of colic is essential for helping to guide owners’ decisions on costly treatments, and for assessing the continuous development of standards of care in the hospital. This study aimed to determine the short-term survival rates of horses admitted with colic to the University Hospital for Large Animals (UHLA), University of Copenhagen, Denmark, from 2010 to 2018, and to compare these to a previous local study as well as recent, comparable international studies.

Short-term survival rates were calculated for horses grouped by treatment (surgical, medical) and diseases. Results were compared to the selected studies using Chi-square tests.

**Results:**

A total of 1752 horses were admitted with colic during the period, of which 355 were excluded for reasons such as economic restrictions or immediate euthanasia. Short-term survival of the remaining 1397 cases was significantly higher (83.0% (95% CI 81.1–85.0%)) than a previous local study (76%) and a recent Dutch study (80%). Medical treatment was carried out in 77.1% of cases, and surgery in 22.9% of the cases. Short-term survival for medically (89.7%) and surgically (60.6%) treated horses was significantly higher in the present study compared to the previous study (87% and 42%, respectively), but was similar to that found in the Dutch study. Significantly fewer horses were euthanised during surgery than in the previous study (17.2 vs. 40%), and significantly more horses recovered from surgery (79.1 vs. 56%). Short-term survival rate of surgically treated horses (60.6%) did not differ from other European studies (55–62%).

**Conclusions:**

Short-term survival rates have increased since the previous study at UHLA, mainly due to a decrease in intraoperative euthanasia. Survival rates in this study are similar to those found in recent comparable colic studies.

**Supplementary Information:**

The online version contains supplementary material available at 10.1186/s13028-022-00631-4.

## Background

Colic is one of the most common health problems and causes of mortality in horses [1–4]. Colic is characterised by abdominal pain and covers a wide range of diseases related to the abdomen and gastrointestinal tract [5–7]. In general, colic accounts for 13.8–35.2% of all hospitalised horses, and surgical treatment is needed in approximately 19.0–43.3% of hospitalised cases [1, 5, 6, 8–10]. Short-term survival (STS) has been defined as survival until discharge from the hospital [11] and the overall STS for all colic cases at equine hospitals has been reported at 68–70% [5, 6, 12]*.* However, the STS varies according to the specific diagnosis, severity, and required treatment [5, 6, 13]. Few studies have researched the STS of medically treated colic in hospital populations [5, 6], whereas the STS of surgically treated cases has been studied more thoroughly [5, 6, 13–15]. Colic is a high-ranking health concern for horse owners, and both medical and surgical treatment can have considerable economic costs, even when treatment is futile [2, 14, 16–18]. Therefore, up-to-date and disease-specific knowledge, as well as evidence-based guidance with regard to the prognosis are of great importance before a decision is made to continue with either surgery or intensive medical treatment [18–22].

The aim of the present study was to determine the overall, medical, and surgical STS of colic cases admitted to the University Hospital for Large Animals (UHLA), University of Copenhagen, Denmark, during the period 2010–2018. Furthermore, this study evaluated the development in STS over time and in comparison to other university hospitals. The three main hypotheses were: (1) STS for both medically and surgically treated colic cases was higher in the present study (2010–2018) than in the previous study at UHLA (2000–2009) [5]; (2) STS for colic cases at UHLA increased within the period examined in the present study (2010–2018), with a higher STS in the latter half (mid-2014 onwards); (3) STS for colic cases at UHLA is comparable to the STS published from equine hospitals in The Netherlands, Norway and Finland [12, 13, 15, 22].

## Methods

### Study population and variables

Data were obtained from medical records of adult (> 1 year old) horses admitted to UHLA for colic between 1st January 2010 and 31st December 2018. Demographic data (gender, age and breed), history, disease process, diagnosis, treatment and outcome were entered into an Access database. Colic was defined as acute abdominal pain [1, 5]. Cases were excluded if they were: dead or moribund upon arrival, diagnosed with colic of extra-enteral origin, or if colic developed during hospitalisation for another disease. Cases where the owner elected euthanasia due to financial or personal concerns other than poor prognosis were also excluded.

Cases with chronic or recurrent colic were included only if they were hospitalised due to an acute episode of colic. In cases of multiple colic episodes or readmission within 30 days, only the first case was included. When repeated episodes were more than 30 days but less than 6 months apart, subsequent cases were only included if the diagnosis was unrelated to the first episode. Similarly, for horses undergoing colic surgery more than once during the same period of hospitalisation, only the first surgery was included. Cases were categorised as surgical when surgery was performed at any point during hospitalisation, and medical when no surgery was performed.

Diagnosis was based on records of clinical and laboratory data, as well as surgical and post-mortem examination where available. When no diagnosis could be concluded due to insufficient or contradictory data in the medical records, the diagnosis was set as unknown. For cases with more than one diagnosis, the primary diagnosis was chosen. The primary diagnosis was defined as that most likely to cause the clinical signs present at the time of admission, and with the highest impact on the outcome. Diagnosis was recorded by anatomical location (e.g., stomach, small intestine, and cecum) and disease process (e.g., simple obstruction, strangulating obstruction, entero-colitis), with a specific diagnosis registered where possible (e.g., epiploic foramen entrapment).

The outcome was categorised as either survival until discharge from the hospital or non-survival (death or euthanasia). The surgically treated cases were also categorised by surgical outcome (“Euthanised or died during surgery”, “Euthanised or died in recovery stall”, or “Recovered”). Recovery was defined as the horse walking out of the recovery stall. The recovered surgical cases were further categorised into non-survival and survival until discharge from the hospital. As the present study included only treated cases, the overall STS was compared to that of treated cases in other studies.

### Statistical methods

Data analysis was performed using R studio version 1.2.5019 for Windows 10 [23] and Microsoft Excel 2016. Numerical data were tested for normality using the D’Agostino-Pearson omnibus test.

Descriptive parameters were evaluated non-statistically*.* Survival rates and the distribution of breeds, diagnoses and treatments were calculated using Pivot Tables in Microsoft Excel 2016. Annual STS was calculated to evaluate progress over time. Furthermore, data were split into two equal time periods to evaluate the significance of progress within the study period. A Chi-square test was used to compare STS results from two time periods—1st January 2010 to 30th June 2014 (Period A) and 1st July 2014 to 31st December 2018 (Period B)—and to compare the results to other recent studies [12, 13, 24], including the previous study at UHLA [5]. The level of significance was set to P < 0.05.

## Results

**Fig. 1:**
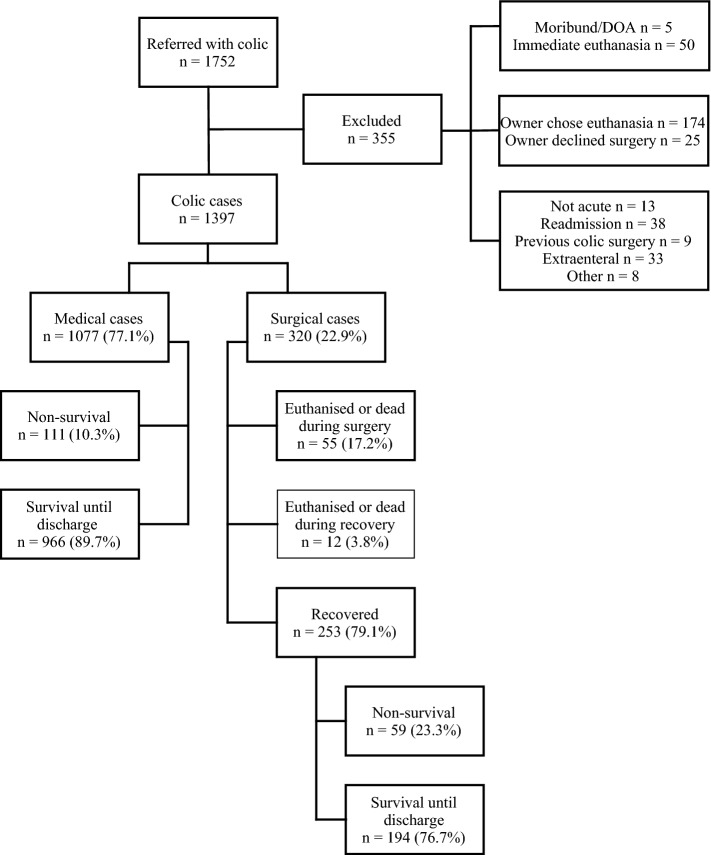
Overview of included and excluded cases. A total of 1752 horses were referred with colic, and 355 of these were excluded from the study for various reasons, resulting in a total of 1397 included horses. The short-term survival rate of medically treated horses was 89.7% (CI 87.9–91.5%). Short-term survival for surgically treated horses was 60.6% (CI 55.3–66.0%). Of those that recovered from surgery, 76.7% survived until discharge from the hospital (CI 71.5–81.9%). *DOA* Dead on arrival

In total, 1752 horses over 1 year old were referred to UHLA for colic during the study period. A total of 8898 horses were admitted to UHLA over the same period, giving an overall colic incidence of 19.7% (95% Confidence Interval (CI) 18.9–20.5%). The total number of colic cases in the database before exclusion (n = 1752) was used, since the 8898 cases included both new admissions and readmissions, as did the total number of horses in the database before exclusion. For this STS study, 355 horses were excluded, resulting in 1397 remaining cases (Fig. 1). Demographic data are given in Additional file 1.

The overall STS was 83.0% (CI 81.1–85.0%), as 1160 out of 1397 horses survived until discharge from the hospital, while 237 were euthanised or died during hospitalisation. Medical treatment was performed in 77.1% (CI 74.9–79.3%) of the cases and surgical treatment in 22.9% (CI 20.7–25.1%) of the cases (Fig. 1). The STS was significantly (P = 0.001) lower in period A (1st January 2010 to 30th June 2014) (78.8%; CI 75.2–82.3%) than in period B (1st July 2014 to 31st December 2018) (85.5%; CI 83.2–87.8%).

The distribution of medically and surgically treated cases did not differ from the previous local study [5] (P = 0.7) nor the Dutch study [12] (P = 0.6), as shown in Table 1. The STS of all treated, medically treated, and surgically treated horses increased significantly from the previous local study [5], but did not differ from that of other recent European studies [12, 13, 24] (Table 1).

**Table 1:** Distribution of treatment and short-term survival rate for the present and comparable studies

Country	Period	Cases (n)	Included	Excluded	Treatments (%)		Short-term survival (%)				
					Medical	Surgical	Overall	Treated	Medical	Surgical	Recovered
(Present study)	2010–2018	1397	All colic cases	Owner declined surgery/treatment, second hospitalization, relaparotomy	77.1	22.9		83	89.7	60.6	76.7
Denmark [5]	2000–2009	1588	All colic cases	Second hospitalization, relaparotomy	76	24	68	76**	87*	42**	75
The Netherlands [12] (Boom)	2012–2013	311	All colic cases	–	76	24	70	80	86	60	
The Netherlands [15] (Loon)	2013–2016	283	Laparotomies	–						59	79.5
Norway [13] (Wormstrand)	2005–2011	297	Laparotomies	Euthanized due to injuries in recovery stall, relaparotomy						55	74
Finland [24] Immonen	2006–2012	236	Laparotomies	–						62	75

The column “Overall” shows the short-term survival for all cases included in the study. Treated cases excludes immediately euthanised cases. Recovered cases includes surgical cases that were not euthanised or did not die during surgery or recovery, but instead walked out of the recovery box alive. Some values in this table were calculated by the authors of the present study based on values from the original articles

*P < 0.05, **P < 0.01

The ascending colon was the most commonly affected portion of the intestine (n = 749; 53.6%; CI 51.0–56.2%). The small intestine was affected in 15.0% (n = 209; CI 13.1–16.8%) of cases in the present study. Anatomical location could not be determined in 17.1% of cases (n = 239; CI 15.1–19.1%). The most prevalent disease process was simple obstruction (n = 604; 43.2%; CI 36.3–41.4%; Table 2). An overview of the specific diagnoses of obstruction in the ascending colon and strangulations of the small intestine can be seen in Table 3.

**Table 2:** Distribution of treatment and outcome for disease process and affected portion for all 1397 horses

Diagnosis	Total		Total medical		Medical		Total surgical		Surgical	
					Survival				Survival	
	n	%^†^	n	%	n	%	n	%	n	%
**Simple obstruction**	**604**	**43.2**	**507**	**83.9**	**492**	**97.0**	**97**	**16.1**	**79**	**81.4**
Ascending colon	502	35.9	427	85.1	420	98.4	75	14.9	60	80.0
Small intestine	35	2.5	18	51.4	18	100.0	17	48.6	16	–
Cecum	26	1.9	23	88.5	20	87.0	3	–	2	–
Stomach	23	1.6	22	95.7	21	95.5	1	–	0	–
Descending colon	15	–	14	–	10	–	1	–	1	–
Rectum	2	–	2	–	2	–	0	–	0	–
Unknown	1	–	1	–	1	–	0	–	0	–
**Unknown**	**227**	**16.2**	**222**	**97.8**	**216**	**97.3**	**5**	**–**	**4**	**–**
**Acute entero-colitis**	**198**	**14.2**	**183**	**92.4**	**125**	**68.3**	**15**	**–**	**7**	**–**
Ascending colon	152	10.9	148	97.4	102	68.9	4	–	1	–
Small intestine	43	3.1	32	74.4	20	62.5	11	–	6	–
Rectum	2	–	0	–	2	–	0	–	0	–
Unknown	1	–	0	–	1	–	0	–	0	–
**Strangulating obstruction**	**158**	**11.3**	**2**	**–**	0	–	156	98.7	84	53.8
Small intestine	108	7.7	1	–	-	–	107	99.1	61	57.0
Ascending colon	46	3.3	1	–	-	–	45	97.8	20	44.4
Cecum	3	–	0	–	-	–	3	–	2	–
Descending colon	1	–	0	–	-	–	1	–	1	–
**Tympany**	**54**	**3.9**	**49**	**90.7**	49	100.0	5	–	4	–
**Gastric Ulcer**	47	3.4	46	97.9	45	97.8	1	–	0	–
**Non-strangulating intestinal infarction**	**27**	**1.9**	**3**	**–**	**0**	**–**	**24**	**88.9**	**10**	**–**
**Peritonitis**	**27**	**1.9**	**22**	**81.5**	**22**	**100.0**	**5**	**–**	**5**	**–**
**Perforation**	**15**	**–**	**10**	**–**	**0**	**–**	**5**	**–**	**1**	**–**
**Chronic entero-colitis**	**13**	**–**	**13**	**–**	**11**	**–**	**0**	**–**	**0**	**–**
**Neoplasia**	**9**	**–**	**7**	**–**	**0**	**–**	**2**	**–**	**0**	**–**
**Grass sickness**	**6**	**–**	**4**	**–**	**1**	**–**	**2**	**–**	**0**	**–**
**Other**	**5**	**–**	**2**	**–**	**1**	**–**	**3**	**–**	**0**	**–**
**Total**	**1397**	**100.0**	**1077**	**77.1**	**966**	**89.7**	**320**	**22.9**	**194**	**60.6**

The most prevalent disease process was simple obstruction (n = 604). Of these, 507 were medically treated and 97 were surgically treated. Of the medically treated simple obstructions, the survival rate was 97% (n = 492), while the short-term survival for the surgically treated cases was 81.4% (n = 79)

Bold text indicates disease process categories. Bold numbers indicate totals for each disease process category

^†^ % of all cases (n = 1397)

**Table 3:** Distribution of treatment and outcome for selected diagnosis codes

Diagnosis	Total		Total medical		Medical survival		Total surgical		Surgical survival	
	n	%^†^	n	%	n	%	n	%	n	%
Ascending colon—simple obstructions	502	35.9	427	85.1	420	98.4	75	14.9	60	80.0
Impaction colon	237	17.0	224	94.5	219	97.8	13	5.5	10	76.9
Left dorsal displacement	126	9.0	120	95.2	118	98.3	6	4.8	6	100.0
Right dorsal displacement	104	7.4	65	62.5	65	100	39	37.5	29	74.4
Other displacements	26	1.9	16	61.5	16	100	10	–	9	90.0
Retroflexion of pelvic flexure	9	0.6	2	22.2	2	100	7	–	6	85.7
Small intestine—strangulating obstructions	107	7.7	1	–	0	–	106	99.1	60	56.6
Epiploic foramen entrapment	46	3.3	0	–	0	–	46	100	19	41.3
Other strangulation	28	2.0	0	–	0	–	28	100	21	75.0
Strangulating lipoma	20	1.4	0	–	0	–	20	100	12	60.0
Volvulus	12	0.9	1	8.3	0	–	11	91.7	8	72.7
Intussusception	1	0.1	0	–	0	–	1	100	0	–

Diagnosis codes causing simple obstructions of the ascending colon and strangulating obstructions of the small intestine. For example, impactions caused simple obstructions of the ascending colon in 17.0% (n = 237) of all 1397 horses. Of these 237 horses, 224 were treated medically and 13 were treated surgically. The medically treated impactions causing simple obstructions in the ascending colon had a survival rate of 97.8% (n = 219), while the short-term survival of surgically treated cases was 76.9% (n = 10)

^†^ % of all cases (n = 1397)

## Discussion

This study retrospectively investigated the STS of 1397 horses with colic admitted to the UHLA during the period 2010–2018.

The overall STS was 83.0%. This rate was significantly higher than the STS of treated horses found in a previous study at UHLA [5], but similar to that of another recent study [12]. However, it is necessary to be cautious when comparing survival rates from different studies and hospitals due to differences in study design and inclusion/exclusion criteria. The overall STS could not be compared directly, as unlike other studies, the present study did not include horses euthanised without treatment.

### Treatments

The STS of medically treated horses is generally higher than that of surgically treated horses due to the generally lower severity of medically treated disease processes such as simple obstructions compared to surgically treated disease processes such as strangulating obstructions [5, 6, 12, 25]. The distribution of diagnoses and treatments at a hospital or within a study population will therefore have a considerable effect on the total STS. In the present study, horses receiving no treatment were excluded prior to evaluation of STS, in contrast to other studies [5, 13]. To help with comparisons, distributions and STS were calculated and compared only to treated cases from other studies (Table 1). The distribution of treatments (77.1% medical and 22.9% surgical) did not differ among studies, which supports the comparison of overall STS.

The owner’s ethical beliefs about animal suffering often influence the decision of whether or not to euthanise. These beliefs vary from country to country, which may complicate the comparison of survival rates if these cases are included in survival studies. In Israel, for instance, euthanasia is rare due to religious beliefs, while it is more common in Scandinavia [5, 13, 26]. The present study excluded all horses euthanised for reasons other than poor prognosis, such as concomitant disease or financial or personal concerns, in order to limit this bias and to obtain results that can be used to give realistic advice to owners. Including these cases in an outcome analysis may create a vicious cycle, as the choice to euthanise will lower survival rates, resulting in even more owners choosing to euthanise [5]. Excluding these cases will minimise the effect of the owners’ concerns, thus providing more accurate survival rates. The other studies did not exclude these cases [5, 12].

The STS of medically treated horses was significantly higher (P = 0.02) in the present (89.7%) compared to the previous (86.6%) study at UHLA [5] (Table 1). This suggests an improvement over time, which may indicate advances in the diagnostics, level of care and treatment of medical colic. UHLA moved to new premises in February 2008, and the improved facilities might also have increased the survival rate of medical and surgical cases. It is, however, also possible that the horses were less severely affected, perhaps due to an earlier referral.

While many studies have evaluated the survival rates of horses with colic requiring surgery, only a limited number have reported the survival of medical colic cases. The reason may be that a diagnosis is often given in cases requiring surgical treatment, whereas in many medical cases, the diagnosis remains unknown. Furthermore, the medical treatment of colic is based on analgesics and fluids, and is therefore largely symptomatic and supportive rather than a specific intervention, so the effects are less easily investigated than surgical techniques [27]. A variety of different drugs and treatments are often used, and protocols may differ among hospitals and attending veterinarians. Although the overall STS of medically treated colic approaches 90% in the present study, further improvements should still be sought, especially for severe and specific diseases such as enterocolitis, where the STS remains relatively low (67%).

The STS of surgically treated horses in the present study (60.6%) was significantly (P < 0.001) higher than in the previous study at UHLA [5] (42%), but similar to other studies (Table 1) [12, 13, 15, 24]. The improvement in overall STS at UHLA was largely due to a decrease in the proportion of horses that died or were euthanised during surgery (17.2%), which was significantly lower than the previous study [5] (39.8%; P < 0.001) and a recent Norwegian study [14] (26.2%; P < 0.05), but similar to a Finnish study [24] (17.4%; P > 0.9). This indicates an improvement over time at UHLA and other hospitals, yet differences among hospitals, studies, and horse populations, may also influence these results. It is likely that the difference in the number of horses euthanised intraoperatively could be related to the general advance in surgeons’ skill and attitude in terms of attempting surgical procedures that were previously not considered possible, such as intestinal resection [28]. In addition, early referral and surgical intervention have been found to improve survival rates [29–31]. However, as the duration of colic pre-referral was not always available from the medical records used in the present study, the effect of this could not be determined. The proportion of horses that were euthanised or died in the recovery stall (3.8%) in the present study did not differ significantly from the previous study at UHLA [5] (3.9%; P = 0.9). Likewise, the STS of horses that recovered from surgery (76.7%) did not differ significantly from the previous UHLA study (74.6%, P = 0.6) [5].

### Diagnoses

The distribution of diagnoses in the present study differed somewhat from comparable studies, mainly due to differences in categorisation and exclusion criteria impeding comparison. In the present study, lesions were categorised by the disease process, anatomical location in the gastrointestinal tract and specific diagnosis code, resulting in a large number of categories. However, the categorisation used in the present study was considered more applicable in clinical practice and allowed the data to be included in more suitable categories. However, due to the complicated nature of equine colic and the lack of consensus in terms of categorisation, it is difficult to know which cases were included in specific categories in other studies, thus complicating comparisons further.

Survival rates have been shown to vary according to both anatomical location and the character of the disease. Since small intestinal lesions are often associated with a lower survival rate than large intestinal lesions, higher proportions of small intestinal lesions will result in a lower overall STS [5, 32, 33]. In the present study, small and large intestinal lesions accounted for 15.0% and 59.5% of cases, with an STS of 60.8% and 84.5%, respectively. In comparison, Christophersen et al*.* [5] found small intestinal lesions in 14% of cases and large intestinal lesions in 54% of cases, of which 34% and 78% survived. The present study therefore found a similar proportion of cases but with higher survival rates compared to the previous study at UHLA [5], especially for small intestinal lesions.

### Improvement in short term survival over time

The STS of 78.8% in Period A was significantly lower than the 85.5% observed in Period B (P = 0.001), which suggests an improvement over time. Comparing two periods within the same study limits the number of external factors that can influence survival rates (e.g., exclusion criteria and categorisation). The study period is relatively long, and there have been many advances in clinical and laboratory examinations, as well as treatment protocols over the course of the study. One measure that is likely to have improved decision making and survival rates is the evaluation of peritoneal fluid lactate, which has improved the diagnosis of ischemic lesions and early decision making in relation to surgery [28, 34–40]. Peritoneal fluid lactate measurement was introduced as a standard protocol in cases of colic at UHLA in July 2013. Another modality that may have contributed to improving diagnostics is the increasing use of transabdominal ultrasonography. Ultrasonography can be useful in diagnosing intraabdominal abnormalities like dilated small intestines in horses with small intestinal strangulating obstructions before they can be palpated rectally, thereby allowing for earlier decisions on surgery [41–46]*.* However, the present study did not interpret these factors and thus cannot determine the effect of each of these advances.

The use of head and tail rope-assisted recovery was implemented at UHLA in 2014 with the aim of reducing complications during recovery, and subsequently reducing mortality in surgical patients. The present study did find an apparent reduction in the proportion of horses that died or were euthanised during recovery from the period spanning 2010–2013 (5.1%, n = 7) to the period spanning 2014–2018 (2.7%, n = 5), however this reduction was not significant (P = 0.5). A recent paper from our hospital evaluated the effect of head and tail rope-assisted recovery and indicated reduced complication rates. In that study, both emergency abdominal surgery (P = 0.004) and a longer duration of surgery (P = 0.0001) increased the risk of fatal complications. Assisted recovery significantly (P = 0.02) reduced the risk of fatal complications after colic surgery [47].

## Conclusion

The STS of horses admitted to UHLA has improved over time and is currently similar to that of other recent European STS studies of equine colic. This improvement in the STS of surgically treated horses was largely due to a decrease in the number of horses that died or were euthanised during surgery. This might be due to improved pre-surgical diagnostic evaluation leading to more rapid decisions about surgery, as well as improved surgical skills and positive attitudes among the surgeons performing intestinal resections.

## Supplementary Information

**Additional file 1.** Distribution of age (years), bodyweight (kg), sex, breed and type for all horses referred to the hospital due to colic.

## Data Availability

The datasets used and/or analysed during the current study are available from the corresponding author on reasonable request.
