# Supplementary material for: Short-term survival rates of 1397 horses referred for colic from 2010 to 2018
Source: Acta Vet Scand. 2022 May 7;64:11. doi: 10.1186/s13028-022-00631-4 (PMC9077955; doi:10.1186/s13028-022-00631-4)
Supplement: Supplementary file 1 — Additional file 1. Distribution of age (years), bodyweight (kg), sex, breed and type for all horses referred to the hospital due to colic. [file 13028_2022_631_MOESM1_ESM.docx]

## Additional files

**Additional file 1. Distribution of Age (years), Bodyweight (kg), Sex, Breed and Type for all horses referred to UHLA due to colic.** “Warmblood” includes Danish Warmblood (DV), other Warmbloods, Holsteiner, Oldenburg, Irish Sport Horse, Hannoveranian, Frederiksborger, and Trakehner. “Pony” includes all pony breeds including Icelandic horses. “Other/unknown” includes horses of unknown breed, mixed breed, and breeds represented by fewer than 20 horses, such as Knabstrupper (n=9).

|  | **Min.** | **Median** | **Mean** | **Max.** | **Unknown (n)** |
| --- | --- | --- | --- | --- | --- |
| **Age (years)** | 1 | 10.0 | 10.3 | 34 | 31 |
| **Bodyweight (kg)** | 58 | 492.5 | 510.0 | 850 | 198 |
| **Sex** |  | **n** | **%** |  |  |
| Gelding |  | 930 | 53.1 |  |  |
| Mare |  | 744 | 42.5 |  |  |
| Stallion |  | 74 | 4.2 |  |  |
| Unknown |  | 4 | 0.2 |  |  |
| **Breeds** | **Type** | **n** | **%** |  |  |
| Warmblood | W | 772 | 44 |  |  |
| Icelandic | C | 397 | 23 |  |  |
| Pony | C | 190 | 11 |  |  |
| Coldblood | C | 86 | 5 |  |  |
| Mixed breed | † | 85 | 5 |  |  |
| Other/unknown | † | 73 | 4 |  |  |
| Friesian | W | 36 | 2 |  |  |
| Standardbred | W | 31 | 2 |  |  |
| Thoroughbred | W | 31 | 2 |  |  |
| Arab | W | 26 | 1 |  |  |
| Western | W | 25 | 1 |  |  |
| **Grand Total** |  | **1,752** | **100** |  |  |

W=Warm-blooded, C=Cold-blooded, ^†^May be warm- or cold-blooded, or of unknown type.
